# Pattern formation, ruptures, and repairs in treatments of personality disorders: an idiographic case series study

**DOI:** 10.3389/fnhum.2025.1552895

**Published:** 2025-07-23

**Authors:** Stine S. Høgenhaug, Mickey T. Kongerslev, Franco Orsucci, Giovanna Zimatore, Sune V. Steffensen, Andreas Ekberg, Matteo Campanella, Guenter Schiepek, Gry Kjaersdam Telléus

**Affiliations:** ^1^Clinic North, Psychiatric Hospital, Brønderslev, Denmark; ^2^Department of Clinical Medicine, Faculty of Medicine, Aalborg University, Aalborg, Denmark; ^3^Mental Health Services West, Region Zealand, Slagelse, Denmark; ^4^Norfolk and Suffolk NHS Foundation Trust, Research and Development Hub, Norwich, United Kingdom; ^5^CEMHS: Centre for Excellence in Mental Health Sciences, University of Amsterdam, Amsterdam, Netherlands; ^6^Department of Theoretical and Applied Sciences, eCampus University, Novedrate, Italy; ^7^Centre for Human Interactivity, Department of Culture and Language, University of Southern Denmark, Odense, Denmark; ^8^Danish Institute for Advanced Study, University of Southern Denmark, Odense, Denmark; ^9^College of International Studies, Southwest University, Chongqing, China; ^10^National Advisory Unit Personality Psychiatry, Division Mental Health and Addiction, Oslo University Hospital, Oslo, Norway; ^11^Clinic for Group Therapies and Personality Disorders Nydalen District Psychiatric Center, Oslo University Hospital, Oslo, Norway; ^12^Institute of Synergetics and Psychotherapy Research, Paracelsus Medical University Salzburg, Salzburg, Austria; ^13^Psychiatry, Aalborg University Hospital, Aalborg, Denmark; ^14^Psychology, Department of Communication and Psychology, Aalborg University, Aalborg, Denmark

**Keywords:** interpersonal physiology, process research, recurrence quantification analysis, adherence, alliance, rupture, repair, personality disorder

## Abstract

**Background:**

Any human communication is based on verbal, emotional, and movement patterns that weave within and between conversation partners. Personality disorders (PD), characterized by emotional dysregulation, attachment instability, and impulsivity, present disruptions in the integration of these coordination dynamics influencing alliance formation and outcome. Therapists, regardless of their clinical expertise, often find themselves grappling with the complexities of tailoring PD treatment. The alliance is often challenged by significant tension or breakdowns increasing risk of impaired progress. Thus, this multi-method comparative case series study investigated how four therapists tailored their treatment with four PD patients in a mentalization-based treatment program to identify patterns of interaction that might facilitate or hinder the therapeutic process during sessions characterized by severe disruption.

**Methods:**

The Symptom Checklist (SCL-92) was applied to identify two successful and two unsuccessful PD treatments. The Rupture Resolution Rating System-Revised was used to detect sessions with rupture frequency peaks in each treatment case. Therapist adherence and competence were assessed with the Mentalization-Based Therapy Adherence and Competence Scale. Heart rate patterns were calculated with cross-recurrence quantification analysis to examine synchronization. An interpretative phenomenological analysis examined the therapeutic process, in addition to quantitative measures.

**Results:**

In sessions with increased rupture frequency, therapists had difficulties managing ruptures and struggled to tailor their treatments no matter the treatment outcome and therapist experience level. Therapists showed high contribution to confrontation ruptures, low adherence and competence ratings, decreased ability to stimulate a mentalizing environment, and inattentiveness to the patients' mental and emotional states during rupture management. Interestingly, more positive heart rate recurrence correlations were identified in sessions from successful treatments showing different regulatory patterns in rupture peak sessions from good vs. poor outcome treatments.

**Discussion:**

Our results make a significant contribution to psychotherapy research by offering a multifaceted perspective on how dynamical alliance processes might foster or hinder the therapeutic process. The clinical implications of low adherence, therapist strategic competence, and increased HR synchronization between therapist and patient in rupture intense sessions are discussed.

## 1 Introduction

Regardless of extensive knowledge that psychotherapy works, the underlying mechanisms of change remain unknown (Wampold and Imel, 2015). Increased interest has evolved in the examination of within-session dynamics and of *how* the therapeutic process unfolds on a moment-to-moment basis to determine specific factors important for the therapeutic process and progress (Kleinbub, 2017; Lambert, 2013). To understand the underlying mechanisms of change, it is not enough to examine the dynamics of good clinical practice. It is crucial to get a deeper understanding of the processes of failures, including missed opportunities to identify and repair them, as this might constitute detrimental effects in psychotherapy (De Felice et al., 2019; Oasi and Werbart, 2020). Special consideration has been given to the investigation of ruptures, understood as episodes of breakdowns or strains between the patient and therapist during a therapeutic encounter (Safran and Kraus, 2014). Ruptures might appear in a direct manner, manifested as *confrontation ruptures* in the form of direct complaints about the therapist or patient, their activities, or the progress of treatment. Furthermore, ruptures may be more subtle in the form of *withdrawal ruptures*, including minimal responses, the masking of experiences, or appeasing behavior. *Repair* is the re-establishment of mutual collaboration and the rebuilding of a therapeutic bond (Eubanks et al., 2015). Proper management of ruptures is associated with better alliance quality and outcome, while poor management of ruptures has repeatedly been linked to poor outcome and lower alliance quality (Eubanks et al., 2018).

The alliance is commonly referred to as an intersubjective activity that includes mutual agreement on, and collaborative negotiation of, goals and tasks of treatment, as well as the formation and maintenance of a relational bond (Bordin, 1979). Treatments of personality disorders (PDs) pay special attention to the therapeutic alliance, and a robust alliance may be perceived as progress in itself (Fonagy et al., 2023). PDs are characterized by insecure attachment, emotion dysfunction, and impaired mentalization, referring to the capacity to understand the mental states of oneself and others in relation to thoughts, feelings, intentions, and motivations (Bateman et al., 2018). Indeed, such interpersonal difficulties challenge the establishment of a relational bond and the joint meaning-making processes of negotiating goals and tasks of treatment. There is evidence that ruptures are more frequent and intense in PD treatments compared to other psychiatric disorders and in non-clinical samples (Gersh et al., 2017; Schenk et al., 2020). Studies of rupture trajectories in PD treatments display a tendency for ruptures to emerge non-linearly with single rupture peak sessions (inverted V-shape) or phases of significant rupture occurrence (inverted U-shape) (Schenk et al., 2019). These results indicate, that in PD treatments the negotiation process is, at times, more dysregulated and characterized by significant higher rupture frequency increasing the risk of collaborative breakdowns (Schenk et al., 2019; Stiles et al., 2004). Thus, in PD treatments clinicians should expect periods of substantial tension, which makes demands according to their abilities to tailor their treatments under severe pressure. Studies highlight how phases of destabilization or dysfunction may have the potential for new pattern formation, the gaining of new corrective experiences, and increased outcome if navigated properly (Eubanks et al., 2018; Schenk et al., 2020; Schiepek and Pincus, 2023). These results emphasize the importance of deepening our understandings of rupture management during significant rupture occurrence in PD treatments to help identify how therapist might hinder or improve the collaborative process when facing severe disruption (Safran and Muran, 1996). Such knowledge could enlighten what might be important for clinicians to be aware of and in future work help guide interventions targeting resolution processes when dealing with more destabilized periods of the treatment process (Samstag et al., 2003).

Research indicates that therapists, no matter their level of clinical expertise, struggle to recognize and address ruptures in psychotherapy leaving many ruptures unhandled and unspoken influencing alliance quality and outcome (Hill et al., 2001). Previous results show that therapists' rigid and inflexible adherence to manuals negatively impacts the quality of the alliance (Castonguay et al., 2010a,b). Too-complex interventions, as well as the increased use of transference interventions, have been found to cause misattunement, as the therapist risks missing out on the patients' experiences (Castonguay et al., 1996; Piper et al., 1991). Finally, therapists' inappropriate use of silence, hostility, intrusive behavior, and defensive or controlling interventions have been found to cause harmful effects on collaboration (Ackerman and Hilsenroth, 2001; Chen et al., 2020; Lingiardi et al., 2018; Talbot et al., 2019). In contrast, addressing ruptures with a therapeutic attitude of warmth, empathy, authenticity, openness, flexibility, curiosity, honesty, trustworthiness, confidence, respectfulness, responsivity, relational attunement, a not-knowing stance, non-defensiveness, validation, acknowledgment of one's own contributions to ruptures, noting past successful behaviors, and accurate interpretations are associated with a higher alliance quality (Safran et al., 2001).

The alliance is widely known to predict a larger variance in outcome than specific techniques or therapeutic strategies (Fluckiger et al., 2018). Nevertheless, therapeutic strategies and competencies are often still examined in relation to the association between adherence to specific treatment manuals and outcome (Laska et al., 2014). In the treatment of PDs, mentalization-based treatment (MBT) is one of the most investigated evidence-based manuals structured to target core aspects of PD, including insecure attachment, emotion dysregulation, and interpersonal difficulties (Bateman et al., 2018; Storebø et al., 2020). Studies examining the impact of MBT adherence in relation to outcome indicate mixed results (Luyten et al., 2024; Moller et al., 2017; Nissen-Lie et al., 2023; Vogt and Norman, 2019), calling for further examination of the interplay between alliance processes, therapeutic adherence, strategy, and competencies. Additionally, knowing that specific techniques are either found to improve or impair outcomes does not provide evidence of *how* and *why* they work. Process research on a multimodal level is warranted to better understand the complexity of what drives therapeutic collaborations forward.

This study adopted an embodied perspective, examining how the therapeutic process is shaped and transformed through multimodal, interactional dynamics, both at explicit and implicit levels, taking into account both verbal content and arousal levels (Kleinbub, 2017; Marci and Riess, 2009; Palmieri et al., 2018). Change is facilitated through a multidimensional process of co-creating meaning and regulating arousal, where each part's actions are deeply influenced by the previous moves of the other. This interaction occurs on multiple levels as they collaborate toward their specific goals of treatment (Muntigl and Horvath, 2023; Mylona et al., 2022). Although science requires the examination of quantifiable, explicit, and observable alliance processes, the application of new multidimensional approaches allows for the emergence and investigation of implicit interactional features, defined as more automatic, unconscious, fast, and not explicitly stated ways of engaging. The use of new approaches are advancing rapidly, revealing numerous possibilities for integrating methods from different research areas to deepen our understanding of change mechanism (Koole et al., 2020; Orsucci, 2016). The field is still in its exploratory phase but holds the potential to help clinicans identify crucial interactional processes which could help guide them to be better able to adapt therapeutic strategies to their patients' individual needs in specific contexts (Nyman-Salonen et al., 2022).

In this study, implicit interactional dynamics in the patient-therapist dyads are examined with heart rate (HR) analysis (Daros and Williams, 2019). HR is related to two complementary synergetic systems: the sympathetic nervous system, representing fight and flight responses that help individuals take action when needed, and the parasympathetic nervous system, representing, among others, states of resting, eating, and sexual arousal (Rajendra Acharya et al., 2006). HR measures are commonly used to calculate heart rate variability (HRV), and the distance RR, defined as the time between heartbeats, which has been shown to be a well-validated biomarker of emotion regulation processes both in healthy adults (Holzman and Bridgett, 2017), in PDs (Wainsztein et al., 2020), and in psychopathology in general (Beauchaine and Thayer, 2015). Evolving evidence is beginning to show associations between increased HR activation and rupture and repair processes (Dimascio et al., 1957; Mylona and Avdi, 2021; Mylona et al., 2022). Additionally, HR synchrony, defined as a shared temporal organization of physiological signals of interacting people (Kleinbub, 2017), has been identified to play crucial functionalities, including emotion regulation, trust, security, empathy, and meaning-making during ruptures and repairs in psychotherapy (Høgenhaug et al., 2024a). Historically, HR synchrony has been described as an ancient survival mechanism, where infants are dependent on their caregivers to attune and help regulate their physiological behavior. Synchronization provides an environmental input for the child to develop self-regulation strategies, and relational skills placing synchrony as a core mechanism in all normal developmental processes (Feldman, 2012; Orsucci et al., 2016). In psychotherapy, higher synchrony between patients and therapists have been associated with higher alliance quality and better outcome (Kleinbub, 2017; Ramseyer and Tschacher, 2011). In PD treatments, synchrony has been proposed to help therapists and patients deepen their emotional bond and increase alliance quality. Studies suggest that PD patients may expand their emotion regulation capacities by initially “leaning” on the interactional process, which might over time be internalized as self-regulatory strategies (Feldman, 2012). Additionally, synchrony has been proposed to increase patients' abilities to explore and process their emotional and mental states (Bar-Kalifa et al., 2023). Prior results indicate how investigating HR synchrony in moments of tension might provide innovative knowledge of ways in which therapists can either help or fail to help patients work with the ability to regulate emotions when facing difficulties (Dimascio et al., 1957; Voutilainen et al., 2018). However, the field is still scattered, with a high degree of inconsistencies and calls for further investigation according to the clinical implications of HR synchronization between patients and therapists (Mylona and Avdi, 2021; Paulick et al., 2018).

This multi-method case series study aimed to investigate therapeutic strategies and competence to increase knowlegde of helpful and harmful alliance features when facing severe disruption. To accomplish this goal, in-session interactional dynamics in rupture peak sessions were compared in two succesful vs. two unsuccesful psychotherapy treatments for PDs. The examination entailed connecting ratings of ruptures and repairs, ratings of manual adherence and strategic competence, and HR time series measurements from both therapists and patients analyzed by correlation and non-linear techniques to measure synchronization. Two research questions (RQs) were examined: (1) How do therapists manage alliance ruptures in PD treatment in sessions including high rupture frequency from good vs. poor outcome cases? (2) How are therapists and patients heart rates synchronized in high frequency rupture sessions?

The hypothesis according to RQ1 was that therapists in good outcome cases would be better able to tailor their treatments (i.e. better adherence and competence ratings and better repair) during intensive rupture frequency sessions. Regarding RQ2, the hypothesis was that therapists in good outcome cases would be more synchronized with their patients compared to therapists in poor outcome cases.

Idiographic examinations of ruptures are limited but highly warranted to help identify crucial in-session factors driving or hindering developmental growth (Castonguay et al., 2010a; Knox, 2019). Thus, a comparative idiographic case series study design was conducted. Although nomothetic approaches are valuable for highlighting general associations between therapist and patient characteristics, alliance quality, and treatment outcomes, they can fall short in capturing the dynamic and fluctuating nature of within-session interactions (Gelo et al., 2020). For instance, global correlates may not capture individual differences and might fail to identify important context-specific patterns (Kramer et al., 2022). Idiographic and multi-method examinations of micro-processes can help to identify and address alliance weaknesses, enhancing our understanding of the mutual contributions and responsibilities during alliance formation (Samstag et al., 1998).

## 2 Materials and methods

This study used data drawn from a larger research project named *The Ecology of Psychotherapy: Integrating Cognition, Language and Emotion* (EPICLE). The data were collected in an outpatient clinic specialized in using MBT for PDs and anxiety disorders (ADs) in a psychiatric hospital in Denmark. The overall aim of the project was to examine change processes in psychotherapy. Thirty patients were enrolled in EPICLE between 2016 and 2020.

### 2.1 Ethics

This project was conducted in accordance with the Helsinki Declaration and prior to enrollment, the project was reported to the Danish National Committee on Health Research Ethics and to the Data Inspectorate, Journal No. 2015-57-0008. The patients and therapists were orally informed about the nature of the project, and all gave written consent to participate. They did not receive compensation, and participation did not influence their treatment.

### 2.2 Selection of participants

All patients were assessed with the Present State Examination Questionnaire (Wing et al., 1974) and the DSM 5's Structured Clinical Interview (SCID-II) (First et al., 1995) before entering EPICLE. Inclusion criteria were any of the following diagnoses: anxiety disorder, obsessive compulsive disorder, or PD. Exclusion criteria were active abuse, autism, or intellectual disability. After initial assessment, patients who met the inclusion criteria were invited to participate in EPICLE.

To give indication of pre-post outcome, the Symptom Checklist (SCL-92) was applied (Derogatis and Cleary, 1977). The SCL-92 is a well-validated self-report questionnaire used to examine affective and psychological distress. It consists of 92 questions and provides an overall Global Severity Index score (GSI). Questions are answered on a 5-point Likert scale, ranging from 0 (*not at all*) to 4 (*extremely*). Raw cut-offs for the GSI are 1.08 for females and 0.87 for males, as identified in normative Danish samples (Olsen et al., 2006). Higher scores indicate greater symptom severity.

**Figure 1:**
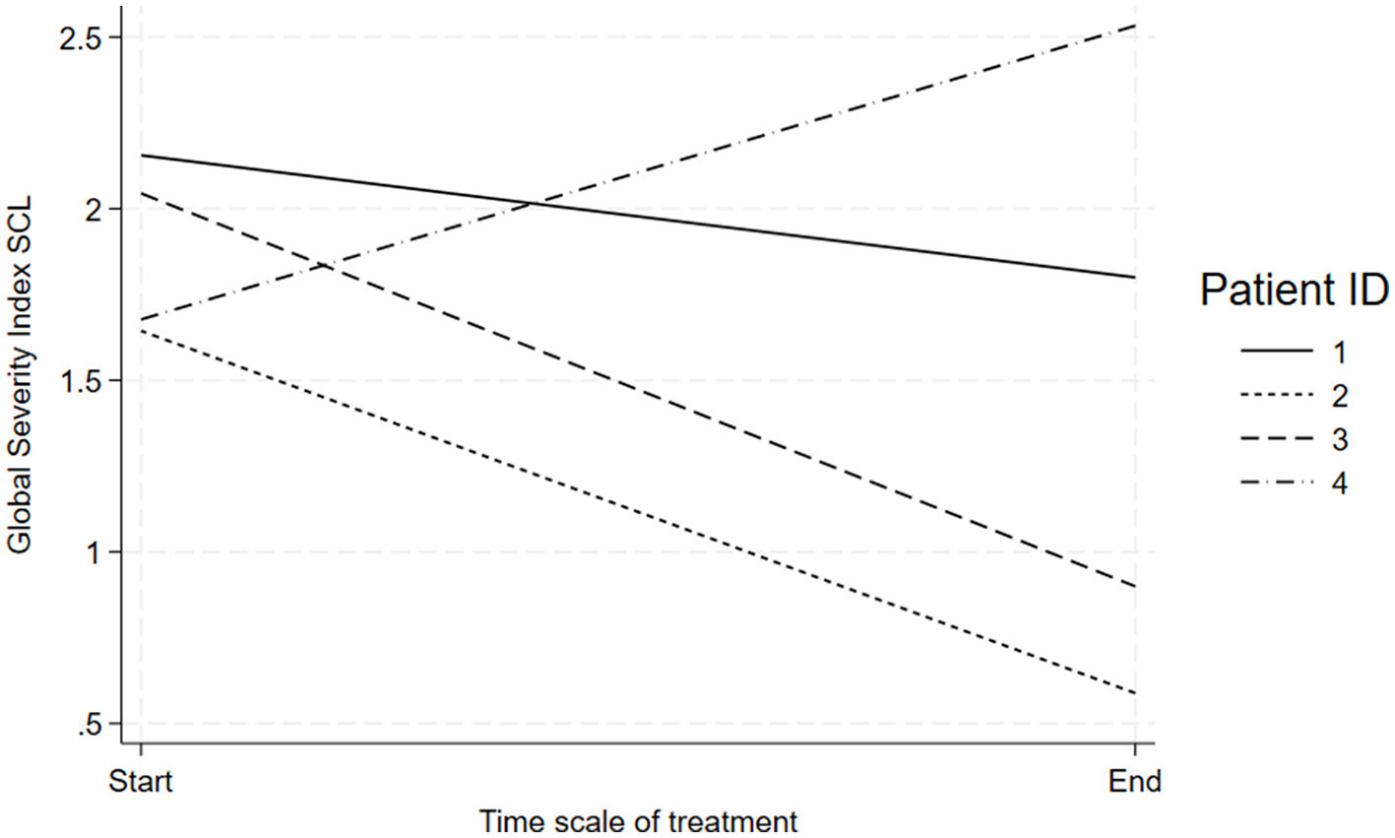
SCL-92 Progress From Pre-Post Treatment. Note: Line graph showing the Global Severity Index SCL over the treatment timeline from start to end for four patients.

All patients enrolled in the EPICLE project, who received a primary diagnosis of PD, and who had completed the pre-post questionnaires were examined to select the cases for this study (*N* = 10); three patients were excluded because of missing data (i.e., electronic or measurement failures). From the seven remaining patients, four patients were chosen for further analysis based on the principle of maximum variation (Suri, 2011). The selection of patients with good and poor outcomes was done through estimation of the relative change [(post-pre)/pre] on the SCL-92 scale. Subsequently, two patients with the largest percentage reduction and two patients with the smallest percentage reduction were chosen as those eligible for further study (Figure 1). Three patients received 1 year of individual therapy (patients 1, 2, 4), while the fourth patient received 1.5 years of individual treatment (patient 3). Patients were treated by four psychotherapists with different levels of clinical expertise (Table 1).

**Table 1:** SCL-92 overview of outcomes and participants.

**Patient ID**	**Diagnosis**	**Therapist expertise (years)**	**SCL baseline**	**SCL treatment end**	**SCL % change**	**Total number of sessions**
1	Mixed PD, including traits from APD and BPD	5–10	2.16	1.80	−16.5	40
2	APD and BPD	5–10	1.64	0.59	−64.2	34
3	BPD	>20	2.04	0.90	−56.0	60
4	BPD and ED	>20	1.68	2.53	51.0	44

APD, avoidant personality disorder; BPD, borderline personality disorder; ED, eating disorder. All patients identified as females and belonged to the same age group (18–25 years).

### 2.3 Alliance rupture resolution rating manual

Alliance rupture and repair were assessed with the Rupture Resolution Rating System-Revised (3RS) (Eubanks and Muran, 2022). On the 3RS, video recorded sessions are divided into 5-min segments and rated on a 5-point Likert-scale from 1 (*no rupture*) to 5 (*severe rupture)* and 1 *(no repair)* to 5 *(successful repair*). The 3RS is an observer-based rating system coding for events of confrontation ruptures, withdrawal ruptures, and repair strategies for both patients and therapists.

### 2.4 MBT—Adherence and quality

The well-validated Mentalization Based Therapy Adherence and Competence Scale (MBT-ACS) was administered to examine therapist adherence and competence (Karterud et al., 2013). The MBT-ACS consists of 17 items for methodological compliance and therapeutic competence, according to the MBT manual. Adherence is assessed by the number of therapist interventions that fall under each item of the scale. Competence is assessed for all items on a Likert scale, ranging from *not at all* (1) to *extensively* (7). An overall adherence and competence score is provided per session. A cut-off score had been established at 4 and above, indicating acceptable MBT fidelity (Simonsen et al., 2019).

### 2.5 Physiological arousal in the output of heart rate

HR was measured by attaching a pulse transducer to the index finger on both patient and therapist to monitor blood volume pulse (BVP), using the photoplethysmogram (PPG) method. Data collection was done through the BioNomadix system of wearable devices, with a sampling frequenc of 125 Hz.

### 2.6 Qualitative analysis

To examine therapists' negotiations of ruptures and repairs beyond quantitative operationalizations, an interpretative phenomenological analysis (IPA) was conducted (Eatough and Smith, 2017). IPA has previously shown efficiency in the analysis of specific phenomena in the field of health psychology and allows in-depth examinations of phenomena like rupture, repair, competence, and responsivity, which makes it a suitable tool to bridge different concepts and fields.

### 2.7 Procedures

The procedure was a multi-layered process which included quantitative ratings of ruptures and repairs, adherence, and competence ratings, HR analysis, and qualitative within-session analysis. Examiations were performed seperately in different research teams, before finally integrating and connecting results to be able to detect a multifaceted representation of the session characteristics.

### 2.8 Alliance rupture resolution

Sessions were coded for ruptures and repairs based on video and audio material. Research Electronic Data Capture (REDCap) (Patridge and Bardyn, 2018), a web-based application designed to capture data for clinical research, was applied to collect the rated data. Sessions were coded for segments of confrontation/withdrawal ruptures (ratings of 3–5) or no confrontation/withdrawal ruptures (ratings of 1–2), and repairs (ratings from 3 to 5) or no repairs (ratings from 1 to 2). Rupture and repair segments were summed up per session. Prior to coding the sessions, two raters were trained for ~50 h, including an introduction course by the developers of the manual, theory, and rating of training videos until sufficient interrater agreement was reached. During the rating period, the raters met weekly for consensus ratings. Interrater agreement was calculated using the weighted kappa method (Cohen, 1968). It showed excellent agreement in relation to withdrawal ruptures (0.836), substantial agreement in relation to confrontational ruptures (0.689), and excellent agreement in relation to repair strategies (0.839).

### 2.9 Session selection

**Figure 2:**
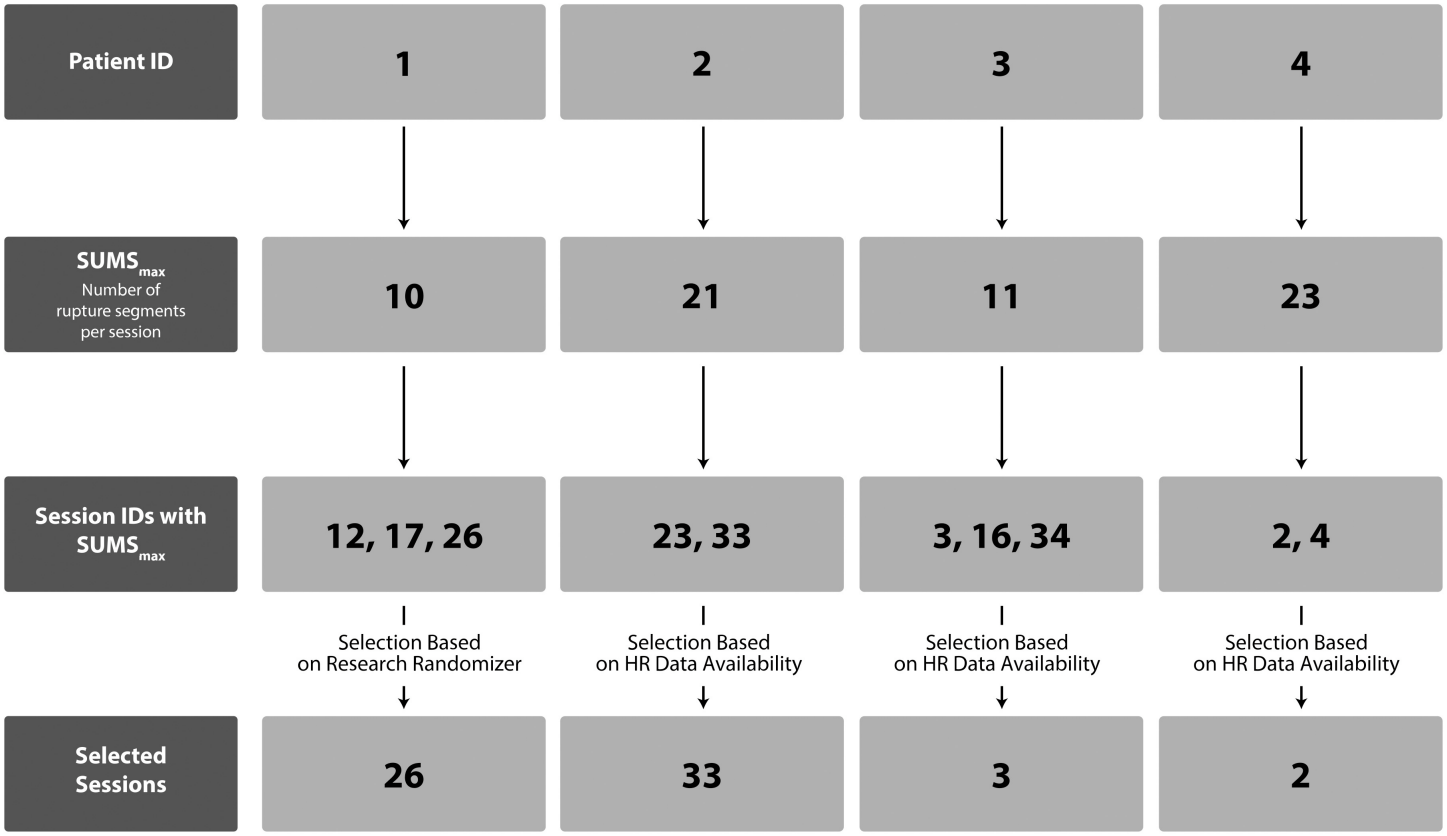
Flow Chart of Session Selection. Note: Flowchart depicting session selection for patients with the maximum number of rupture segments. SUMSmax = maximum sum score of rupture segments for patients and therapists within sessions.

A total of 136 sessions were coded with the 3RS of the four treatments; 42 sessions were not coded because of missing video material or technical issues with sound or picture. To be able to conduct detailed, multi-method investigations of rupture management in rupture peak sessions from good and poor outcome treatments, the session selection in each case was based on the maximum sum score of rupture frequency ratings for both patient and therapist per session. Sessions with frequent rupture occurrence have the potential to reveal different patterns of rupture navigation within sessions representing severe distress. Such knowledge might provide important insight into how patients and therapists move in and out of cycles of ruptures and repairs in critical periods during their treatment process. This was hypothesized to provide excellent material for in-depth analysis on multiple levels of interaction to identify key alliance features in highly intense situations that might hinder or facilitate therapeutic growth. Additionally, session selection was based on the available physiological data of more than two-thirds of the signal per session. One session from each dyad was selected to be able to both compare sessions from good and poor outcome treatments and to able to examine potential patterns between sessions from good outcome treatments as well as poor outcome treatments. More sessions were identified in each case with the same maximum rupture sum score. For patients 2–4, only one of the sessions met the criteria regarding HR data availability, while a randomized session selection tool, the Research Randomizer (Urbaniak and Plous, 2013), was used to choose the session for further analysis for patient 1 (Figure 2).

### 2.10 MBT adherence and competence

The MBT-ACS was assessed with session-recorded videos and transcriptions. The ratings were conducted at the Quality Laboratory of Psychotherapy at Oslo University Hospital, Norway.^1^ Ratings were both done as an overall estimate per session and as specific intervention ratings within the session transcripts to be able to determine specific patterns of interaction. The adherence scale had previously shown good interrater agreement (Simonsen et al., 2019), and the Quality Laboratory conducts regular reliability testing for its raters to ensure consistent reliability in ratings. Due to the exploratory nature of this study, one experienced rater (the sixth author, AE) was found suitable for rating (Simonsen et al., 2019).

### 2.11 Physiological arousal in the output of heart rate

The dynamic patterns of HR were examined per patient-therapist dyad in each session. The RR intervals that were used represent the HR-interpolated RR intervals. To obtain these intervals, peak detection and interpolation were applied to the PPG data, using a Hamming window of 60 s with an overlap of 20 s; time resolutions were 125 ms/sample. HR time series are commonly evaluated using non-linear time-domain approaches, feature-based time-domain methods, and frequency-domain methods (Høgenhaug et al., 2024; Rajendra Acharya et al., 2006; Serantoni et al., 2022; Zimatore et al., 2024). In this study, the non-linear approach of recurrence quantification analysis (RQA) was applied to give indication about the autonomic nervous system's dynamics and regulatory processes between the patients and their therapists. CROSS-RQA was used to identify sequences of synchronized HR recurrence patterns between the patient and therapist within the sessions (emb = 7, Euclidean distance, mean dist, cut-off = 15, line = 9) [a more detailed description of the HR analysis can be found in Høgenhaug et al. (2024b) and in Webber and Marwan (2015)]. The Pearson correlation coefficient r between RQA measures was reckoned to describe patient and therapist alliance.

### 2.12 Qualitative analyses

The qualitative procedure was performed based on verbatim transcripts and video material of each session. The analysis was led by the first author in collaboration with three research assistants and supervised by the second (MTK), third (SVS), and ninth (GKT) author. Three of the analysists were experienced mental health researchers, while the other analysists had sparse psychiatric experience. Members of the analytical team represented different levels of experience with qualitative analysis, including one expert in qualitative research (SVS). The analytical approach relied on a phenomenological-hermeneutic process of analysis through five steps presented below (Eatough and Smith, 2017). For theme validation consensus evaluations were performed in each step emphasizing reflexivity and researcher subjectivity (Smith et al., 1999).

#### 2.12.1 Analytical procedure conducted in the four sessions, using IPA

Step 1: The first author (SSH) and three research assistants conducted a detailed review of both videos and transcriptions inductively, discussing the content multiple times to ensure a reflexive in-depth process that included multiple perspectives.

Step 2: While reviewing the material again, the first author (SSH) and the three research assistants aimed for a detailed phenomenological examination of the therapeutic process according to the negotiation of ruptures and repairs, therapeutic competencies, and responsivity between patients and therapists.

Step 3: Reemerging themes were discussed by the first author (SSH) and the three research assistants both within each 5-min segment and as an overall impression for each session. This stage of analysis included the identification of characteristic sequential patterns of timing, precision, and adaptation of therapeutic strategies during cycles of ruptures and repairs.

Step 4: Each theme was discussed and interpreted by the first author (SSH), second author (MTK), fourth author (GZ), and ninth author (GKT), incorporating the analysts' reflexivity and shared evaluation of results from the analytical process. A critical evaluation was conducted according to each theme through an iterative process until sufficient agreement was reached according to the definition and naming of the themes. Multiple theories, including alliance theory, mentalization-based theory, and psychoanalytic and attachment theory were applied to explain patterns of interaction. In this discussion, psychodynamic feedback perspectives given by the sixth author (AE) on each session based on the MBT-ACS ratings were included.

Step 5: Based on the prior steps, major recurrent patterns were identified in each session by the first author (SSH) and the three research assistants. Participants quotes were incorporated as illustrative examples to support the results.

## 3 Results

### 3.1 Rupture and repair ratings

Generally, the therapists were found to contribute more to confrontation ruptures compared to withdrawal ruptures. Among significant therapist confrontation markers were directive, controlling, and pressuring behavior, pushing back by rejecting the patients' ideas, and direct complaints about the patient, activities, goals, and progress. Therapists' use of repair strategies included focusing on tasks and goals, invitations to explore ruptures, acknowledging one's own contribution to ruptures, and linking ruptures to interpersonal patterns.

### 3.2 MBT-ACS

All except one of the therapists were rated below average on MBT adherence, while all four therapists were rated below average on competence ratings (Table 2). The one therapist (session 26) rated above average on MBT adherence was, however, unable to achieve a significant impact with interventions due to a lack of responsiveness to the therapeutic process. Overall, therapists tended to focus more on behavior than mental states and were often found to intervene on a too-complex level. They missed several opportunities to engage the patients in mentalizing processes and the sessions were generally found to disrespect fundamental principles of upholding a mentalizing stance.

**Table 2:** MBT-ACS.

**Patient ID**	**Therapist adherence rating**	**Therapist competence rating**
4 (poorest)	3	3
1 (poor)	5	3
2 (good)	2	2
3 (best)	2	3

MBT-ACS ratings. Cut-off: 4 indicated “good enough” adherence and competence. In parenthesis are indicated outcome treatments (see below).

### 3.3 HR synchrony in each therapist-patient dyad

Comparing the Pearson (r) correlation between percentage of recurrence of HR time series in the four cases, the sessions from the “good” and “best” outcome treatments revealed a moderate positive correlation (*r* = 0.43, 0.35, respectively) compared to a negative correlation in the session from the poor outcome treatment (*r* = −0.37), and no correlation in the poorest one (*r* = 0.06). No differences were observed in the linear correlation of HR time series (Pearson *r* < 0.02). An overview of the Pearson (r) correlation between percentage of recurrence of HR time series in the four cases is provided in Table 3. These results may indicate that even when observably misaligned, underlying regulatory mechanisms may still influence the therapeutic process.

**Table 3:** Percentage of cross-correlation in each patient-therapist dyad.

**Outcome**	**Patient ID**	**Session**	**Total session time (min)**	r^*^(rec)	***r*- (HR)**
Poorest	4	2	52	0.06	0.012
Poor	1	26	56	−0.37	−0.018
Good	3	3	60	**0.43**	−0.019
Best	2	33	62	**0.35**	0.017

^*^Pearson correlation coefficient *r* of percentage of recurrence of two time series of patient and therapist in each session in 10 non-superimposed windows. In the last column, the same essences (<2%) of correlation of the couple of entire HR time series can be observed. The bold values are the positive moderate pearson correlation coefficient r of percentage of recurrence of the two time series of patient and therapist in the sessions from the good outcome cases.

All sessions were examined according to the maximum occurrence of cross-recurrence correlation between patients and therapists within sessions to give indication of synchronization tendencies per patient-therapist dyad. Results indicate that in the best and good outcome cases, the time of the maximum of the cross-recurrence was located earlier compared to the poor and poorest outcome cases (Table 4).

**Table 4:** Cross-RQA within sessions.

**Windows**	**Time (min)**	**4_Poorest**	**1_Poor**	**3_Good**	**2_Best**
1	2.1	0.538	6.442	1.01	0.326
2	6.3	1.934	1.23	2.816	0.76
3	10.4	1.952	2.987	2.072	1.28
**4**	14.6	3.931	2.103	2.583	**2.986**
5	18.8	2.392	6.59	2.413	0.33
**6**	22.9	5.144	1.973	**4.667**	1.14
**7**	27.1	2.956	**7.47**	3.604	1.993
8	31.3	0.546	1.583	2.03	1.423
**9**	35.4	**5.911**	2.275	1.469	1.247
10	39.6	2.091	3.469	0.442	1.246

Cross-recurrence in 10 non-superimposed windows of 2,000 points (4.17 min); absolute max of cross-recurrence in bold.

### 3.4 IPA analysis

Three major themes recurred in the IPA analysis across the four sessions.

Theme 1: *Contribution to Ruptures and Repairs*. Based on the well-operationalized concepts of ruptures and repairs in the 3RS (Eubanks and Muran, 2022), this theme concerned the therapist's contribution to tension or breakdowns through directive, controlling, or pressuring behavior, complaints about the patient, process or progress of the treatment, tendencies to be hostile, push back, and defend themselves when challenged. Furthermore, contributions could involve therapists' shutting down, giving minimal response, or using denial, abstract communication, topic shifts, and masking of real experiences. Finally, the theme included the therapists' contribution to restoration of collaboration through focusing on tasks and goals, exploring ruptures, acknowledge their own contribution to ruptures, and linking ruptures to interpersonal patterns.
Theme 2: *Tailoring of Treatment*. It is well-established that rupture and repair strategies in themselves do not account for the influence they may have on therapeutic interactions. The impact of a rupture might be high or low, depending on the strategic competencies of the therapist in relation to tailoring the treatment to specific patients and contexts. Strategic competencies include precision, timing, and relevance of an intervention, which partially overlap with the competence scores on the MBT-ACS (Karterud et al., 2013). Grounded in the competence ratings made by author AE, this theme examined the therapists timing, precision, and adjustment of intervention complexity to the situation and the patient. The aim of this theme is to give indication of effective strategic competencies vs. ineffective or even harmful strategic competencies when managing ruptures.
Theme 3: *Lack of mentalizing the patient*. Based on the concept of mentalization (Fonagy and Allison, 2013), the third theme concerned the therapist's ability to mentalize the patient's mental and emotional states during ruptures. Mentalizing the patient involves the therapist's ability to pay attention to, validate, acknowledge, be curious and open, and invite for exploration about the patients' feelings, thoughts, bodily experiences, intentions, motivations, needs, desires, and longings.

### 3.5 Case presentations

In the next section, the four sessions are presented in relation to each theme to broaden the understanding of the context-specific management of rupture peak sessions in PD treatments.

#### 3.5.1 Patient 4 (poorest): “Your problem is…”

The main topic in this session was trying to come to an agreement about a common understanding of the patient's pathology. Special attention throughout the session fell on the attempt to understand the meaning of the patient's tendency to binge-eat and throw up afterwards. At the beginning of the session, the therapist suggested that whenever the patient felt lonely, she binge-ate and threw up to regulate her emotions. Her behavior made her relatives concerned and often resulted in getting attention from them, which made her feel less alone. Several times the patient reacted to the therapist's interpretations with direct complaints. For example, she stated: “*Last time you said I only threw up to get attention; this is too simple of a way to understand my problems*.” She also showed signs of disconnecting from the discussion, replying to the therapist's questions with: “*I don't know*,” or by giving minimal responses or showing appeasing behavior, saying: “*Maybe I will drop it then […] if you say it does not make sense (smiles)*.” At session's end, the therapist directly asked the patient: “*Do you feel understood by me?*” and the patient replied, “*No*,” followed by retreat, stating that she wished she could express herself more clearly so the therapist would understand the complexity of her problems better.

##### 3.5.1.1 Contribution to ruptures and repairs

Several times the therapist offered directive explanations of how to understand the patient's problem. For instance, stating: “*So, your main problem is that you feel lonely and abandoned*.” Although the patient showed withdrawal by laughing, masking her emotions, giving minimal responses, and showed appeasing behavior, the therapist kept elaborating this understanding and kept criticizing the patient's attempt to manage her problem in a directive and controlling manner stating: “*but, this is stupid [making plans on how to be able to binge-eat when hospitalized]. You are hospitalized because you need to stop binge-eating. Then it does not make sense to be hospitalized.”* The therapist's confrontative behavior was found to increase mixed ruptures from the patient, including direct confrontation about not feeling understood, followed by retreat. Halfway through the session, the therapist made several attempts to repair. Repair strategies included focusing on tasks and goals, and acknowledgment of the therapist's own contribution to ruptures stating: “*I can tell, that the statement I made last time offended you a lot. I am sorry about that.”* The therapist also invited for exploration of their relationship in the here and now: “*How was it to tell me these things, that you were angry with me, for example,”* and validation of the patients thoughts and emotions: “*I do understand, that you are angry with me,”* which at times were found to regulate tension and engage the patient in elaboration for a while. Unfortunately, interventions were made without an in-depth working through of the ruptures, leaving the session without proper repair.

##### 3.5.1.2 Tailoring of treatment

Overall, the therapist struggled to uphold a mentalizing stance of curiosity, openness, warmth, and not-knowing. The therapist was found to use long, confrontative, and explanatory interventions, without adapting the interventions to the patient's level of mentalization. The therapist tended to make efforts to engage the patient in their understanding, often at too complex of a level for the patient to understand. For example, by stating: “*so you had an aunt who gave you some of what your parents didn't have time to give you. Because I think I was sitting, thinking that you were feeling very lonely, or somewhat lonely. We all need attention, and I think the eating disorder has different functionalities, a way of holding something down, because when you don't eat then the restlessness comes, also a way of getting attention. I think, when you get suicidal, then it is because you get desperate, and you get desperate when something is in the air, let's say, something that threatens you with becoming alone*,” and in another example: “*And you had an aunt, whom you were very attached to, so she died, and your feelings got worse.”* The patient mainly replied to these interpretations saying: “*I don't know,” “hm,”* and “*maybe,”* indicating withdrawal and disengagement. When the patient did engage in the process the therapist invited for exploration, but quickly became explanatory again about how the therapist understood the problem. Thus, the tailoring of treatment indicated a lack of adapting interventions to the patient in the situation and indicated an impaired ability to engage the patient successfully in a mentalizing process about the goals and tasks of treatment.

##### 3.5.1.3 Lack of mentalizing the patient

Although showing occasional warmth, curiosity, and empathy, the therapist generally appeared caught up in their own meaning-making process, while deemphasizing the mind of the patient and her ongoing attempts to show her perspectives. For instance, the therapist stated in a rather directive manner: “*We agree that your problem is that your parents had too much work, so they did not have time for you, right? So, you were alone feeling lonely and abandoned, right? So, your main problem is feeling lonely*.” The patient responded with: “*hm*” and “*I don't know*” after each “*right*,” visibly not engaging in the process. The therapist kept pushing, and the patient ended up saying, “*I do not think I am lonely*.” Instead of being curious about the perspective of the patient, the therapist replied stating: “*Are you not? To me it sounds like, you are lonely. You use all your time alone to binge eat. You visit your mother, and she does not have time for your visit*.” Again, the patient tried to engage in the process stating, that the mother did have time. However, instead of being curious and open to the patients understanding, the therapist overruled the statement of the patient and gave another example of the patient's loneliness from hospitalization resulting in increased tension.

#### 3.5.2 Patient 1 (poor): “What we know about you”

The main topic of this session was the patient's lack of engagement in group therapy. During the last group session, another patient announced a pregnancy, which made the patient jealous and sad. The patient did not say anything during the group session but withdrew instead. Throughout the individual session, the patient and the therapist discussed whether it would be possible for the patient to break her pattern of withdrawal and speak her mind in the group next week. The patient explained how she had also talked to her partner about the benefits of breaking her pattern of withdrawal. The therapist asked what she thought about this goal, and the patient replied: “*It [the goal] is a little bit our common goal, but to be honest, it is mostly my partner's*.” Thus, the therapist and patient struggled to get on the same page regarding the goals and tasks of treatment, resulting in ending the session with the patient confirming to speak her mind in group but indicating appeasing behavior and missing full agreement on if and how they should work with her pattern of withdrawal.

##### 3.5.2.1 Therapist contribution to ruptures and repairs

The therapist was found to confront the patient numerous times while discussing goals and tasks of treatment, for example stating, “*What we know about you is that when something is difficult you react by turning everything inwards, leading to feelings of being stupid, not good enough, and failure, which we have discussed and observed on several occasions. When we have seen this pattern, there is something beneath the surface you have not taken care of, and you attend therapy as you want to change so you do not have to feel this way. This, I would like to help you with. But I cannot do it as long as you keep doing the same things*.” And: “*If you keep repeating the same patterns, then you do not benefit from the last part of the individual treatment […] then you are shutting it down, the work, because you did work until now. But in this situation, you have another choice. What do you want.”* The patient mainly responded to the therapist's interventions by withdrawing saying “*I don't know,”* confirming to the therapist's suggestions, and stating: “*I just want to make everyone happy.”* Multiple times the patient's withdrawal led to further explanatory and confrontative behavior from the therapist resulting in increased rupture occurrence.

##### 3.5.2.2 Tailoring of treatment

The therapist managed to somewhat uphold a mentalizing stance with warmth, validation, authenticity, and empathy most of the session, creating a supportive therapeutic climate. However, the interventions were characterized by a high degree of psychoeducation about the patient's primary pattern of withdrawal and explanations regarding the goals of group therapy in relation to the patient's difficulties. The interventions were often long and complex (i.e. the two examples above), and the patient often answered in a withdrawing manner and seemed to have a hard time following the therapist's rationales. The therapist was periodically attentive to their own contribution to the patient's withdrawal, saying, “*Does that make sense?*” but missed several opportunities to engage the patient in a mentalizing process as the therapist quickly turned to further psychoeducation instead of staying curious and inviting for a mutual meaning-making process. What seemed to keep the alliance somewhat balanced and at times seemed to engage the patient in the process were interventions concerning affect integration, where the therapist was attentive to the patient's behavior in the here and now, and the use of interventions that invited the patient to explore her nonverbal behavior: “*Where is it placed in your body [anger]”* and “*do you feel it right now [the anger]?”*.

##### 3.5.2.3 Lack of mentalizing the patient

Across the session, it was observed how the therapist was very active, without giving the patient much time to answer or reflect. The therapist made assumptions about the desires and needs of the patient, without fully exploring the patient's mind. For instance, the therapist stated: “*there is some stubbornness. Some will power in you, and you need to use this to act differently. Because the self-blame, and self-criticism and turning it inwards, I think that is what weighs you down. You need some stubbornness and aggression to get out, to do something different, or you will repeat old patterns. You want to do something different, so what are you going to do on Tuesday in group?”* After this intervention, the therapist kept pushing the patient to engage in a process of how to break her pattern of withdrawal in the next group session. The therapist offered solutions on how the patient could practice in the group without giving the patient space to think about if and how she would like to work with her pattern of withdrawal in the group.

#### 3.5.3 Patient 3 (good): “We cannot have a trustful collaboration when you act like this”

The main topic of the session was the patient's experience of being abandoned and feeling misunderstood by her relatives. She experienced increasing suicidal thoughts and had threatened on several occasions to act on her thoughts. In therapy, she said she had just lost a relative and complained about her boyfriend not giving her enough attention in the process of the loss. She stated that she tried to talk to her boyfriend, which resulted in a major conflict where the patient ended up making suicidal threats, and the boyfriend gave her sleeping pills to avoid a suicide attempt. She still experienced suicidal thoughts and was making threats in the session about not being able to promise she would not act on her thoughts before the next session. The last part of the session included multiple ruptures, while the patient and therapist tried to negotiate a plan for the patient not to act on her suicidal thoughts.

##### 3.5.3.1 Therapist contribution to ruptures and repairs

Initially, the session was characterized by the patient's withdrawal, masking her emotions, laughing, and shifting topic when asked to elaborate and explore her emotional distress. Halfway into the session, confrontation ruptures increased for both the patient and the therapist while negotiating a plan for the patient not to attempt suicide. The therapist showed signs of hostility and control at times in a sarcastic tone, complaining about the patient's behavior, saying: “*We have a relationship, and it does not work if I cannot count on you being here next week […]. This does not work; you cannot have relationships with other people like this, that maybe our appointment next week is not going to happen because you killed yourself […]. This would affect others; it affects me*.” and “*you need to do something [when having suicidal thoughts], because it's no use, it is not possible for it to be something like 50–50. It's not, it does not work. It's not being honest with yourself. You must be.”* The patient responded with confrontation ruptures including this comment: “*This is a contract I can't sign […]. I do not want to sit on the floor thinking, now I let everybody down […] then I am never going to get back up again*.” The patient's confrontation was followed by an increase in the therapist's confrontation ruptures while they discussed the patient's threatening behavior. While confronting the patient, the therapist displayed periods of trying to repair by explaining task rationales, and revealing the motivation for wanting to collaborate with the patient on a common project stating: “*my intention is to help you get better, and be better able to manage things differently, as I don't find what you are doing now helpful for you,”* and some degree of acknowledgment of own contribution to ruptures saying: “*I could imagine you are angry that you have the feeling that I don't understand you, that I don't acknowledge that you have been feeling bad.”* However, by the end of the session, the ruptures remained unresolved. The patient ended up agreeing not to act on her thoughts until their next session. In a retreating fashion, she said: “*Until now, I am still here, aren't I?*” while smiling in a sarcastic way.

##### 3.5.3.2 Tailoring of treatment

While negotiating ruptures, the therapist intervened multiple times in a sarcastic tone, with a lack of warmth, used very few validating statements, and used long complex interventions. The interventions were often made in an overruling, hostile, directive, and controlling tone focusing on what the patient needed to do, rather than trying to engage the patient in a mentalizing process about how to understand her suicidal triggers and current emotional distress. For example stating: “*[…] So, we have to find something, so that you have an emergency solution when you get like that, so that you can either say, okay, I'll get my partner, he'll help me, or I'll call the Psychiatric Emergency Room, if there's anyone, so you'll do everything you can to work on that when you get like that, because you have to at least give this a chance, you have to, that the treatment also involves, okay, I'll give this a chance. That means that the next year we have together, I must be able to count on you working hard and intensely to keep yourself alive. That you don't give up once a month, because it's unbearable to have a relationship with people who do that.”* Thus, the tailoring of treatment was not found stimulate mentalization, and lacked adaptation to the mentalizing level of the patient in the context.

##### 3.5.3.3 Lack of mentalizing the patient

The therapist's interventions were mainly focused on behavior instead of mental states. The therapist periodically invited the patient to explore her feelings, but quickly started to justify their own point of view instead of trying to understand the mind of the patient. For example, the therapist asked: “*What are you feeling right now,”* the patient replied, “*I don't know”* followed by “*I am angry I don't know why.”* The therapist validated the anger, but then turned to suggest how to understand why the patient was angry instead of inviting for further elaboration from the patient. The patient rejected the therapist's interpretation, resulting in the therapist stating: “*do you know what I think. I think you are really poor at letting yourself feel the way you do, because you explain every time, that if you have feelings, you do not understand, it is not ok to feel them. And I think you have a long practice ahead of you where you need to let yourself feel bad sometimes. Let yourself be sad, let yourself be angry […].”* Additionally, it was observed, how the therapist started taking the perspectives of the patient's relatives instead of being curious about the patient's mind. For instance, stating: “*So, it becomes his [boyfriend] responsibility [calling the emergency center]. That is a big responsibility to put on your partner.”* The patient responded with trying to defend herself resulting in a discussion about whose responsibility it was to call the emergency center when having suicidal thoughts.

#### 3.5.4 Patient 2 (best): “You did complain the same way about engaging in individual therapy as you are complaining now about group. This looks like avoidance to me, you see?”

The main subject in this session was the patient's ending of individual treatment and lack of engagement in group therapy. The patient stated major disapproval of group therapy throughout the session. For instance, saying: “*I really struggle to engage in the group […]. I do not feel comfortable, and I have struggled to find my place in the group*.” In the session the patient and therapist discussed how the patient had felt overlooked and had felt used as a tool by the therapist (who was also the therapist in the group) during the last group session to engage another patient in a mentalizing process. The patient confronted the therapist stating: “*I ended group last time feeling used by you as a tool to help A. to see something or to get better […]. My role was mainly being a tool*.” The patient kept addressing concerns about the group. She did not find the group helpful and was afraid of getting “worse” if she kept attending group sessions after the end of individual treatment.

##### 3.5.4.1 Therapist contribution to rupture and repair

Several times the therapist confronted the patient about not engaging in the group saying: “*you withdraw, at least from giving it importance […],”* and “*If you want to stop group therapy now, for example, my colleagues, because that's how we work, we work in teams, will be very critical about […] Why? Why doesn't she want what helps her?”* The patient engaged in the conversation, both showing confrontation ruptures, including complaining about the therapist and the treatment rationales, and withdrawal ruptures, using laughter and appeasing behavior. When confronted by the patient the therapist often identified this and acknowledged their own contribution to the rupture right away stating: “*I could certainly follow you on that, and it was also clear that it was difficult for you, because it was me who continued what A. said without taking a critical stance on what she had actually said*” and“*Nor was it considered that what you wanted to talk about was how scared you were. I agree with that.”* Additionally, the therapist tried to repair by inviting the patient to explore the ruptures in the here and now in the session. Additionally, what appeared to balance the confrontative nature of the conversation was that the patient was continuously found to engage in a meaning-making process according to the therapist's rationales even when criticized by the therapist. For example, the patient engaged in discussing her attendance in group therapy stating: “*because you referred me to group therapy because you thought I would get something out of it, but I doubt it. So that's why I'm saying it now instead of telling you that I don't plan on coming anymore*.”

##### 3.5.4.2 Tailoring of treatment

Although missing out on several opportunities to engage the patient in a mentalizing process, the therapist succeeded in staying warm, validating, and empathic most of the session. Also, the therapist seemed to manage a “good enough” tailoring of the complexity of her interventions in correspondence with the patient's level of mentalization. However, a characteristic of this session was, that the therapist did the work for the patient, meaning that instead of collaborating in a mentalizing manner to co-create meaning, the therapist was very active, in an overruling and non-mentalizing manner, in attempts to make the patient understand the therapist's perspectives. The therapist mainly responded to the patient's concerns in a psychoeducational manner, providing explanations about the task rationales stating: “*We have great experience with the combination of group and individual treatment, and we often experience that when individual treatment ends, then patients start using the group in a better way*,” or by using multiple interpretations including: “*And I think, that was the decisive factor in you losing yourself in it, because you trust me so much, and trust that I know, not best, but good. So, if I use A's words, it must be because your perception is wrong.”* Hence, the therapist did not give the patient much time to elaborate but often interrupted the patient to elaborate their own perspective.

##### 3.5.4.3 Lack of mentalizing the patient

Overall, the therapist's very active, explanatory, interpretative, and psychoeducational style did not leave much space for the patient to explore her own thoughts, feelings, intentions, and needs during the session. The therapist often invited for exploration, but then turned to make claims about the patient's mind instead of staying curious. Consequently, the patient ended the session complaining about how they used their time during session: “*I have sometimes, and especially the last few times, been tired of how much time I have spent in here discussing the group, when there are many other things important to me, I also needed to talk about.”* To which the therapist, instead of exploring the patient's perception, replied in a directive manner: “*I think ultimately, it's just about you are feeling like there's not enough time. That you have so much more you want to talk about.”*

## 4 Discussion

The aim of this multi-method study was to increase knowledge of alliance features that might facilitate or hinder therapeutic collaborations during severe disruption in sessions of successful and unsuccessful treatments of patients suffering from PDs. The multi-method approach consisted of rupture and repair ratings, MBT adherence and competence ratings, HR calculation, and a qualitative analysis of the therapeutic interaction. Contrary to our first hypothesis, results show that the therapists, no matter their clinical level of expertise and treatment outcomes, had a hard time managing rupture intense sessions on an observable level of interaction. The therapists' confrontation ruptures were found to contribute negatively to the therapeutic process by increasing tension. Among the unhelpful alliance processes were hostility, sarcasm, lack of warmth, controlling or directive behavior without validating the patient's mind or feelings, too long and complex interventions, lack of agreement on tasks and goals of treatment, and absence of curiosity. These findings reinforce previous results, showing how therapists confrontation ruptures negatively impact alliance processes, and also results showing a more general tendency of therapists to struggle to identify and address ruptures in psychotherapy (Castonguay et al., 2010a).

In this study therapists' tailoring of treatment, was examined by applying the MBT-ACS. Therapists in good outcome cases were hypothesized to be better able to tailor their treatments in sessions revealing severe disruption. However, this was not supported by results as all therapists were found to struggle according to either manual adherence and/or strategic competence performance when under pressure. The main task during therapy sessions viewed through the lens of adherence and competence ratings is for the therapist to maintain a focus on mental states, stimulate a mentalizing process, and challenge or explore impaired mentalization (Karterud et al., 2013). One of the therapists from the poor outcome cases was rated above average on adherence, while the two therapists from the good outcome cases were rated below average on the adherence scale. All therapists were rated below average on the competence ratings. This could raise a question of whether adherence and competence related to a specific method may play a small role in determining change (Webb et al., 2010). Thus, a good treatment process does not necessarily rely on the ability to follow a specific treatment manual (Donkin et al., 2011). Contradicting this argument is that the qualitative analysis uncovered how the treatment process in each session might have improved if the therapists had shown better abilities to stimulate mentalization and uphold a mentalizing stance during their management of ruptures. Therapists' failures to properly practice their strategic competencies were found to increase rupture intensity in all sessions. These results reinforce prior research that has examined the importance of upholding a mentalizing stance during rupture management (Fonagy et al., 2023). As only four sessions were rated, no conclusions can be drawn according to the impact of the therapists' adherence and competence ratings in relation to outcome. Indeed, perhaps the selected sessions in this study do not represent the work in other sessions. The selection of sessions representing maximum rupture frequency most likely influenced the therapists' usual treatment and that the therapists' behavior in other sessions differs significantly from the rupture peak sessions. For example, the therapists might show a better ability to repair ruptures or demonstrate better adherence or competence when under less pressure. Future work should include more sessions that reflect variability in rupture occurrence to increase knowledge of the interplay between rupture and repair processes, adherence, therapeutic strategies, and competence.

The therapists all showed a willingness to explore ruptures and confront patients' dysfunctional patterns, which has previously been proposed as important in facilitating change (Folmo et al., 2019). However, while challenging the patients' dysfunctional patterns, the IPA analysis revealed how all the therapists struggled to pay sufficient attention to the patients' mental and emotional states. The lack of mentalizing the patients' perspectives were identified as a factor increasing strains in all sessions. One hypothetical explanation for the therapists' lack of mentalizing their patients appropriately could be due to transference-countertransference processes. Previous research has found countertransference reactions to be associated with more ruptures and results have found such reactions to be inversely related to outcome, while proper management of countertransference has been found to contribute to resolution and positive outcomes (Hayes et al., 2018). Prior results show, that therapists report strong countertransference reactions of anger, confusion, and anxiety in response to the unpredictable behavior of patients suffering from PD's. Safran et al. (2001) argues that to work effectively with ruptures, therapists must be aware of their own feelings, acknowledging their own subjective perceptions. Several studies have also shown how repair is fostered when therapists shift their focus from their own agenda to attending to the patient's experiences or emotions (Kazantzis et al., 2017). Thus, future studies could include therapist self-reports to give indication of transference-countertransference processes to increase understandings of how these processes might influence the management of severe distress (Tishby and Wiseman, 2014).

### 4.1 Synchronizing on an implicit level

Confirming our second hypothesis, the results showed a moderate positive HR synchrony between patients and therapists in sessions from good outcome cases, while no correlation and a negative correlation were observed in sessions from poor outcome cases. Previous studies have shown physiological synchrony between patients and therapists to be an important marker of adaptive interpersonal co-regulation, and as such an essential process for developing the therapeutic alliance (Orsucci et al., 2016). Findings have repeatedly revealed associations between higher synchrony, higher alliance quality, and increased outcome (Ramseyer and Tschacher, 2011). Hence, as the synchrony values are consistently positive and moderate in successful cases, while being near-zero or negative in the poor cases, results might be hypothesized to suggest that HR synchrony is a measure sensitive to important alliance processes. Notably, HR synchrony alone might not be a definitive marker of therapeutic success but may reflect implicit relational dynamics that differentiate good from poor outcomes. Importantly, the focus in this study was not on the absolute magnitude of r, but on the systematic difference in synchrony between outcome groups. Based on our very small sample size, this is a highly tentative proposal as the Pearson correlation might represent other constructs than the alliance during sessions.

Interestingly, the time of the maximum of the cross-correlations between patients and therapists was found to be inversely proportional to the goodness of the outcome. Multiple studies have revealed an association between increased early treatment synchronization and outcome (Tufekcioglu et al., 2013). However, studies related to the fluctuating nature and impact of high synchronization early within sessions and their impact on a session level is lacking and needed to increase idiographic understandings of the therapeutic process. On a theoretical level it could be hypothesized, that high early synchrony within sessions might reflect increased early alliance quality, which might reduce the negative impact of ruptures later in the session (Schiepek et al., 2023; Schiepek and Pincus, 2023). Nevertheless, much work is still needed examining HR correlation patterns within sessions before any strong conclusions can be drawn in this regard and future studies are recommended to examine within session HR synchrony dynamics.

### 4.2 Early and late assessments of ruptures and repairs

Studies have shown how early disruptions can cause maladaptive alliance dynamics throughout a course of treatment (Muran et al., 2009). Additionally, the preponderance of dropouts occur within the first sessions of treatment, highlighting the importance of establishing a strong early alliance (Samstag et al., 1998). PDs are associated with low mentalizing abilities, which cause more dropouts, making early ruptures even more important to resolve. Two sessions examined the initial treatment process [session 2 (poorest) and 3 (good)]. In both sessions, the influence of the therapists' contribution to ruptures was found to be high and caused increased tension. The findings from session 2 align with previous results, showing an early unresolved rupture session and poor outcome. Contradicting this, results from session 3 showed an early unresolved rupture session and decrease in symptom severity by the end of treatment. This result is encouraging and provides hope for a therapeutic process even after a rough start that includes an unresolved rupture peak in the initial phase of treatment (Stevens et al., 2007). Thus, the alliance does not need to be of high quality in each session throughout the early treatment process. Hence, an unresolved session might very well be resolved in the following sessions or might be used as a psychological subject for therapeutic work in later sessions, which was not examined in this study (Zilcha-Mano et al., 2015).

Two sessions were from the late phase of the treatment process: session 26 (poor) and 33 (best). Studies have suggested that ruptures later in therapy might be opportunities for new corrective experiences (Cirasola et al., 2024; Gersh et al., 2017). In session 26, the patient reacted primarily using withdrawal when the therapist contributed to ruptures. The patient's behavior could reflect a willingness to assert negative feelings of being misunderstood to preserve a pleasant environment, avoid conflict, protect the alliance, and protect the therapist, which could indicate old patterns of behavior rather than a new corrective experience. In session 33, the patient was found to contribute to repair by mentalizing the therapist's perspective without undermining her own perspective, which was found to help regulate tension. The patient's behavior in this session might reflect the manifestation of new behavioral patterns based on her ability to move between mentalizing herself and the therapist in a session reflecting severe distress, which is one of the main goals in PD treatments (Bateman et al., 2018).

### 4.3 Strengths, limitations, and future directions

A strength of this study is that it contributes to psychotherapy research by including a multifaceted understanding of how therapists' navigation of challenging situations might foster or hinder the therapeutic process. Naturally, the results should be read in the light of their limited generalizability, as only four sessions were examined. Thus, no solid conclusions can be drawn from the results of this case-series study. The sessions represented maximum rupture frequency at different times during the treatment process in two good and poor outcome treatments. The selection of the extreme outcome cases and sessions was done to be able to uncover potential significant recurrent patterns of interaction during treatment periods of severe disruption. The results relied on the assumption that these selection criteria would enlighten interactional dynamics between ruptures, tailoring of treatment, and synchronization. However, it is possible that selection of these sessions risk overemphasizing dysfunction, while at the same time underestimating constructive resolution in other sessions. Thus, selection might exaggerate therapists struggling during rupture management. It is possible, that an examination of randomized sessions, sessions with lower rupture occurrence, or sessions within the same period of the treatment process might have revealed different dynamics (Schenk et al., 2019). Moreover, synchronization was only examined by the output of HR. Different nonverbal modalities have been found to operate on different timescales and may serve different functionalities (Orsucci, 2021; Orsucci et al., 2016). Hence, it is possible that other synchronization patterns would appear following the inclusion of other modalities (i.e., skin conductance, oxytocin, movement). It is also probable, that HR patterns observed in this study might not reflect alliance dynamics but may represent different interactional patterns not accounted for in this study as no correlation analysis was performed between ruptures, repairs, and HR synchronization. Additionally, this study did not control for the influence of patient heterogeneity, and therapist effects, which might have affected the current findings. Another possible limitation was that one of the researchers conducting the IPA (first author, SSH) was a specialist in MBT, which could be viewed as a potential bias. Nonetheless, the purpose of IPA is to facilitate insider perspectives while at the same time recognizing that the analysts' subjective perceptions are their primary tool for examination. An analyst's perspective is requisite for interpretation and as such viewed as a strength in relation to some study findings (Larkin and Thompson, 2011). Furthermore, to balance potential bias, the three research assistants and one of the supervisors, who was a specialist in conducting qualitative research (SVS) involved in the analysis had no former clinical experience with MBT.

Future studies are encouraged to include a larger sample size and a greater variance in session selection to increase knowledge of the relationship between rupture management, adherence, competence, and synchronization. This could give a richer and more nuanced picture about helpful and unhelpful alliance processes while managing different rupture frequencies. Such knowledge could in future work help enlighten what might be important to target when training and supervising therapists. Accompanying this perspective is a recommendation to apply a more detailed microanalytical procedure within sessions when studying synchronization. The alliance quality is well known to fluctuate within sessions. Hence, studying micro-processes has the potential to reveal the clinical impact of synchronization patterns between patients and therapists when managing ruptures (Høgenhaug et al., 2024b). Finally, investigations of interpersonal synchrony in different modalities are endorsed to better understand how different constructs might relate to one another, to alliance processes and to outcome. For example, HR is known to offer a reliable measure of the intensity of emotional reaction, but is less effective as a measure of the valence, positive or negative, where other modalities are found to be better suitable (Basu et al., 2016). Therefore, in the future, it could be meaningful to include and compare HR and other modalities for further discrimination and analysis of emotional responses.

## 5 Conclusion

By applying a multi-disciplinary approach, this study showed the potential of bridging concepts of ruptures and repairs, strategic competence, and HR synchrony to gain in-depth insights into helpful and harmful navigation of ruptures on multiple levels of interaction in rupture peak sessions. This study indicates how therapists' contributions to confrontation ruptures, lack of stimulating mentalization, intervening on too complex a level according to the patient in the context, and inattention to the patient's mind with poorly functioning patients lead to increased tension and lack of repair during interaction. The findings appeared consistent at different levels of clinical expertise and at different times in the treatment process when managing sessions representing severe disruption.

Different HR patterns were observed between sessions from good and poor outcome cases. Thus, it is possible that different underlying regulatory mechanisms might have influenced the therapeutic process in each session. This study underlines the need to engage in idiographic context-specific studies, applying a multi-disciplinary method when examining rupture management to create multifaceted understandings of helpful and harmful effects.

## Data Availability

The datasets presented in this article are not readily available because it contains participants identifiable data about patients and therapists. Requests to access the datasets should be directed to s.v.steffensen@sdu.dk.

## Footnotes

^1^Kvalitetslaboratorium for psykoterapi—Oslo universitetssykehus HF.
